# Human leukocyte antigen B*0702 is protective against ocular Stevens–Johnson syndrome/toxic epidermal necrolysis in the UK population

**DOI:** 10.1038/s41598-021-82400-3

**Published:** 2021-02-03

**Authors:** Gibran F. Butt, Ali Hassan, Graham R. Wallace, Shigeru Kinoshita, Sajjad Ahmad, Mayumi Ueta, Saaeha Rauz

**Affiliations:** 1 Academic Unit of Ophthalmology, Birmingham and Midland Eye Centre, Institute of Inflammation and Ageing, College of Medical and Dental Sciences, University of Birmingham, Dudley Road, Birmingham, B18 7QU UK; 2 Birmingham & Midland Eye Centre, Sandwell and West Birmingham Hospitals NHS Trust, Birmingham, UK; 3 Moorfields Eye Hospital NHS Foundation Trust, London, UK; 4 Institute of Ophthalmology, University College London, London, UK; 5 St Paul’s Eye Unit, The Royal Liverpool and Broadgreen University Hospitals NHS Trust, Liverpool, UK; 6 Department of Frontier Medical Science and Technology for Ophthalmology, Kyoto Prefectural University of Medicine, Kyoto, Japan

**Keywords:** Genetic association study, Conjunctival diseases

## Abstract

Stevens–Johnson Syndrome and Toxic Epidermal Necrolysis (SJS/TEN) are part of a disease continuum of vesiculobullous mucocutaneous reactions affecting the skin and mucous membranes including the ocular surface. Manifestations of disease range from mild dry eye to progressive conjunctival cicatrisation, limbal epithelial stem cell failure and corneal blindness. In Far Eastern and South East Asian populations where SJS/TEN is prevalent, numerous human leukocyte antigen (HLA) gene variants at the A, B and C loci have been identified as risk factors for developing SJS/TEN with severe ocular complications (SOC). By contrast, the incidence of SJS/TEN with SOC in European countries is relatively low. To date, ocular SJS/TEN risk altering alleles have not been widely investigated in European populations. In this study, we analysed the association of HLA -A, -B and -C alleles with SJS/TEN in 33 patients residing in the UK with age matched controls. The data showed statistically significant novel negative allele association with HLA-B*0702 and a trend with HLA-C*0702 in the patient group, indicating these alleles are protective. Further characterisation of protective and risk alleles in other ethnic groups is required to fully elucidate the putative role of these alleles in the susceptibility of SJS/TEN with or without severe ocular complications in patients in the UK.

## Introduction

Stevens-Johnson syndrome and Toxic Epidermal Necrolysis (SJS/TEN) are part of a disease spectrum of inflammatory vesicobullous mucocutaneous reactions affecting the skin and mucous membranes resulting in blisters, erosions and loss of tissue. The appearance of typical cutaneous and mucosal lesions is preceded by fever, malaise and upper respiratory tract symptoms^1^. SJS is diagnosed when less than 10% of total body surface area is involved compared to more than 30% in TEN, and an overlapping intermediate form between 10 and 30%. In its most extensive form, the condition can be life threatening. While it is a rare orphan disorder with a minimal estimated incidence of 0.8 per million in the UK^2^, its risk is significantly greater in Asian populations^3^. For example in Japan and Korea the incidence is up to 3.4 and 5.03 per million respectively^4,5^. The acute phase cutaneous desquamation is accompanied by risks of sepsis and multi-organ failure with associated mortality rates of 5% in SJS and up to 30–40% in TEN^6,7^. Although SJS/TEN are largely considered to be acute drug reactions, numerous other precipitants have been identified including bacterial and viral infections^8,9^.

SJS/TEN pathogenesis is characterised by a type IV delayed hypersensitivity reaction against a trigger, with symptoms typically manifesting a few days after exposure^6^. The precipitant antigens are processed and combined with human leucocyte antigens (HLA) by antigen presenting cells (APCs) following which they are externalised on the cell surface^10,11^. Interaction of this complex with a T-cell receptor ultimately leads to CD8+ cell mediated injury through Fas ligand and granulysin release, causing apoptosis in epidermal and mucosal tissues^1,10,11^. In humans the MHC gene complex on chromosome 6 encodes HLA proteins. HLA genes are polymorphic, which enables the immune system to process a wide range of foreign antigens to the T cell receptor. The precise interplay between antigen molecular structure and metabolism, genetic risk factors and T cell clonotypes are all thought to contribute to the risk of developing SJS.

Multiple HLA alleles have been associated with numerous autoimmune diseases as well as SJS/TEN in a variety of population groups. Such associations have also been characterised with ocular SJS/TEN in different ethnic populations, mainly in the Far East Asian populations. Both positive associations (presence of an allele, conferring an increased risk if present) and negative associations (absence of an allele, conferring a risk reduction if present) have been described in the literature^12–29^.

Ocular inflammation is a highly prevalent early feature occurring in over 70% of cases, whilst chronic ocular disease may occur in over 50% of these patients^30,31^. Acute phase ocular surface inflammation may be mild or lead to severe ocular complications (SOC) including severe conjunctivitis, pseudo membranes, forniceal adhesions, and large epithelial defects of the conjunctiva and cornea. Exploring the histories of patients with ocular SJS/TEN, chronic disease is manifest by a spectrum of mild to severe ocular complications, the latter include disabling dry eye with keratinisation, recurrent episodic inflammation, scleritis and progressive conjunctival cicatrisation, limbal epithelial stem cell failure and corneal blindness^32^. There is increasing evidence that genetic and drug associations of SJS/TEN is linked to a distinct severe phenotype of ocular disease^33–36^.

The immunogenetics of ocular SJS/TEN to date, is yet to be studied in a European population. Ocular SJS/TEN is rare in this population compared with South East Asia where there is increasing evidence that the incidence and prevalence might be due to underlying genetic susceptibility. In this study, we therefore characterised HLA class I genes (HLA-A [Gene ID: 3105], HLA-B [Gene ID: 3106], and HLA-C [Gene ID: 3107]) in 33 SJS/TEN patients of diverse ethnic backgrounds residing in the UK presenting at three of the largest dedicated eye centres in the UK, to determine whether any associations exist between these patients’ genotype, phenotype and precipitant for our patients compared with published data from patients in Far East Asia.

## Methods

### Patients and samples

Patients with SJS/TEN attending ophthalmology outpatient clinics were consecutively recruited from the Birmingham & Midland Eye Centre (Birmingham), Moorfields Eye Hospital (London), and St Paul’s Eye Hospital (Liverpool) between 2015 and 2020. Patient were longstanding attendees at their respective clinics and had a biopsy confirmed or clinically confirmed diagnosis made by the referring physicians. Controls subjects were recruiter from cataract pre-operative assessment clinics and hospital departments. All recruits underwent ophthalmic examination and a saliva sample for genetic analysis.

This study was approved by the NHS Research Ethics Committee (LREC ref: 08/H1206/165), institutional review boards of the University of Birmingham and Kyoto Prefectural University of Medicine and was conducted in accordance with the Helsinki Declaration. All recruits provided informed consent.

### HLA genotyping

DNA samples extracted from saliva (Oragene DNA kit; Kyodo International) according to manufacturer’s protocol were analysed for all known possible HLA-A, HLA-B and HLA-C alleles by polymerase chain reaction (PCR), followed by hybridisation using the commercially available sequence-specific oligonucleotide fluorescent probes (Wakunaga Pharmaceutical)^26–29,37,38^. Samples were sequenced for all MHC loci for the genes encoding HLA- A, B and C antigens. Target DNA was PCR amplified with biotinylated primers specifically designed for amplified exons 2 and 3 of HLA-A, HLA-B, and HLA-C genes. The biotinylated polymerase chain reaction product was labelled with phycoerytrin-conjugated streptavidin and immediately examined (Luminex 100; Luminex). Genotype determination and data analysis were performed using WAKFlow typing software (Wakunaga) according to the manufacturer’s instructions.

### Statistical analysis

The frequency of individuals with a given HLA allele termed “carrier frequency” and frequency of the alleles at the population level termed “gene frequency” of individual HLA alleles in our patients and controls was compared. Each allele was considered as an independent variable and odds ratio (OR) and 95% CI were calculated using GraphPad Prism 8.3.0. Significance was assessed using the Fisher exact test.

## Results

Saliva samples from 33 patients with SJS/TEN and 24 healthy controls were genotyped. The study group consisted of 22 (67%) White (White-British 21, White-European 1) patients, the remainder being of Indian-subcontinent (6(18%)), Black-African Caribbean (3(9%)), or mixed (2(9%)), ethnic background. The control group consisted of 15 (65%) White-British, Indian subcontinent 4(17%), Black-African Caribbean 3(13%), and Filipino 1(4%) background. Table 1 summarises the demographic details of the study groups.

**Table 1:** SJS/TEN participant demographics.

	SJS/TEN		Controls
Number recruited	33		23
Age at onset			
Range	4–70		
Age at recruitment			
Mean	47		56
Range	23–77		22–83
Gender			
Female	24 (73%)		19 (79%)
Male	9 (27%)		5 (21%)
Ethnicity			
White British	21 (64%)	White British	15 (65%)
White European	1 (3%)	Caribbean	3 (13%)
Caribbean	3 (9%)	Bengali	2 (9%)
Mixed Black -Caribbean and White	2 (6%)	Indian	1 (4%)
Sri Lankan	1 (3%)	Pakistani	1 (4%)
Indian	5 (15%)	Filipino	1 (4%)
SOC	28 (85%)		
Cold medicine SJS with SOC	8 (24%)		

*SJS/TEN* Stevens–Johnson Syndrome/Toxic Epidermal Necrolysis, *SOC* severe ocular complications.

Twenty-eight patients (85%) had a history of SOC at either acute, chronic or both stages. Most patients had a single precipitant, however eight patients had multiple suspected precipitants. These included cold medicines (8 (24%)), antimicrobials (14 (42%)), antiepileptics (4 (12%)) as well as unknown triggers (9 (27%)). Table 2 summarises the features of the study and control groups.

**Table 2:** SJS/TEN clinical characteristics.

Patient	Sex	Ethnicity	Age at diagnosis (years)	Age at recruitment (years)	Severe ocular complications	Cold medicine	Precipitant
UK SJS 01	F	White British	36	43	N		Penicillin
UK SJS 02	F	White British	40	72	Y		Cotrimoxazole
Uk SJS 03	M	Caribbean	61	77	Y		Unknown
UK SJS 04	F	Indian	30	40	Y		Antimalarial (name unknown)
UK SJS 05	F	White British	39	54	Y	Y	Diclofenac
UK SJS 06	F	Indian	70	72	N		Metronidazole
UK SJS 07	M	White British	23	41	Y		Erythromycin
UK SJS 08	F	White British	17	24	Y	Y	Codeine; paracetamol; deracoxib
UK SJS 09	F	Sri Lankan	4	25	Y		Sulfadoxine & Pyrimethamine
UK SJS 10	F	White British	20	29	Y		Penicillin
UK SJS 11	F	White British	35	50	Y	Y	Ibuprofen
UK SJS 12	F	Mixed race (Black—Caribbean/White)	41	48	Y		Unknown
UK SJS 13	F	Mixed race (Black—Caribbean/White)	23	23	N		Lamotrigine
UK SJS 14	F	White British	58	71	Y		Carbamazepine
UK SJS 15	M	White British	56	51	Y	Y	Aspirin; cylophosphamide; Cotrimoxazole; fluconazole
UK SJS 16	M	Indian	44	44	N	Y	Penicillin; paracetamol; clarithromycin
UK SJS 17	F	White British	56	57	Y		Ciprofloxacin; rifampicin; ertapenem
UK SJS 18	M	Caribbean	31	45	Y	Y	Paracetamol; doxycycline; Beechams*
UK SJS 19	F	White British	30	39	Y		Amoxicillin; Herpes Virus
UK SJS 20	M	White British	9	31	Y		Unknown
UK SJS 21	F	Indian	11	33	Y		Sulfadoxine & Pyrimethamine; ciprofloxacin
UK SJS 22	F	Caribbean	1.5	35	Y		Phenobarbital
UK SJS 23	F	White European	30	34	Y		Lamotrigine
UK SJS 24	F	White British	52	58	Y		Erythromycin
UK SJS 25	F	Indian	45	49	Y		Unknown
UK SJS 26	F	White British	1.5	31	Y		Unknown
UK SJS 27	F	White British	24	45	Y	Y	Ibuprofen
UK SJS 28	F	White British	4	26	Y		MMR vaccine
UK SJS 29	M	White British	49	66	Y		Unknown
UK SJS 30	F	White British	9	63	Y		Unknown
UK SJS 31	M	White British	20	58	Y		Unknown
UK SJS 32	M	White British	6	42	Y	Y	Cold medicine (name unknown)
UK SJS 33	F	White British	52	54	N		Unknown

*Beechams is a part of the Glaxo Smith Klein companies. They produce a number cold & flu formulations which contain a range of ingredients including acetaminophen, non-steroidal anti-inflammatories, caffeine, and phenylephrine. No further precipitant specificity was determinable.

Both patient and control groups were genotyped for HLA-A, -B and -C regions. Of those, statistically significant negative associations of HLA B*0702 and a trend of C*0702 were found, identifying these as possible protective alleles. These results are summarised in Table 3. Similar trends were identified in subgroup analyses of patients with SOC and white patients with SJS/TEN, although these association were not statistically significant. These associations were true for both gene frequency and carrier frequency. Interestingly, this presented as a haplotype in all four SJS/TEN patients with these alleles and seven of the ten controls with these alleles (Supplementary Table 1 and 2).

**Table 3:** UK SJS/TEN patients HLA B*0702 and HLA C*0702 results and sub-group analysis.

	Carrier frequency				Gene frequency			
	No. (%)				No. (%)			
	Cases	Controls	Odds ratio (95% Confidence interval)	Fischer’s exact test	Cases	Controls	Odds ratio (95% Confidence interval)	Fischer’s exact test
All SJS/TEN vs all control	33 (100)	23 (100)			66 (100)	46 (100)		
HLA-B*0702	4	9	0.22 (0.07 to 0.77)	0.026*	4	9	0.27 (0.09 to 0.95)	0.037*
HLA-C*0702	4	8	0.26 (0.08 to 0.98)	0.054	4	8	0.30 (0.10 to 0.98)	0.069
SJS/TEN vs control (White only)	22 (100)	15 (100)			44 (100)	30 (100)		
HLA-B*0702	3	7	0.18 (0.05 to 0.78)	0.056	3	7	0.24 (0.06 to 1.04)	0.079
HLA-C*0702	3	6	0.12 (0.06 to 1.10)	0.117	3	6	0.29 (0.08 to 1.12)	0.146
All SJS/TEN SOC vs all control	28 (100)	23 (100)			56 (100)	46 (100)		
HLA-B*0702	4	9	0.26 (0.08 to 0.95)	0.057	4	9	0.31 (0.10 to 1.15)	0.077
HLA-C*0702	4	8	0.28 (0.08 to 1.01)	0.095	4	8	0.37 (0.12 to 1.17)	0.132
SJS/TEN SOC vs control (White only)	20 (100)	15 (100)			40 (100)	30 (100)		
HLA-B*0702	3	7	0.20 (0.05 to 0.89)	0.062	3	7	0.24 (0.80 to 5.16)	0.235
HLA-C*0702	3	6	0.27 (0.06 to 1.25)	0.129	3	6	0.32 (0.08 to 1.24)	0.158
All SJS/TEN CM-SOC vs all control	8(100)	23(100)			16 (100)	46 (100)		
HLA-B*0702	2	9	0.52 (0.09 to 3.18)	0.676	2	9	0.59 (0.12 to 2.96)	0.713
HLA-C*0702	2	8	0.63 (0.11 to 3.96)	> 0.999	2	8	0.68 (0.13 to 3.65)	> 0.999
SJS/TEN CM-SOC vs control (White only)	6 (100)	15 (100)			12 (100)	30 (100)		
HLA-B*0702	2	7	0.57 (0.09 to 3.82)	0.659	2	7	1.64 (0.26 to 9.31)	0.627
HLA-C*0702	2	6	0.75 (0.12 to 5.17)	> 0.999	2	6	2.00 (0.32 to 11.96)	0.596

*CM* Cold medicine, *SJS/TEN* Stevens–Johnson syndrome/toxic epidermal necrolysis, *SOC* severe ocular complications.

## Discussion

SJS/TEN is a life threatening acute vesiculobullous pathology associated with a broad range of precipitants. Most cases are self-limiting, however the risk of secondary complications and death necessitate specialised management and close monitoring. Ocular involvement, particularly with severe features, is increasingly recognised as a distinct phenotype and warrants early targeted intervention. Chronic ocular features of disease are lifelong and debilitating. Experiential data shows that acute-phase ophthalmic treatment in both adult and paediatric patients, is orientated around minimising ocular surface inflammation, preventing opportunistic infections, and supporting healing to minimise chronic ocular changes. The onset of acute disease and the chronic progression are thought to be associated with underlying genetic susceptibility in populations across the Far East and central Asia^4,5,25^, but little is known regarding the immunogenetics of European populations. Table 4 summarises the known allele associations of ocular SJS/TEN. In this study, we analysed the HLA genotype of SJS/TEN patients residing in the UK and showed novel protective allele associations of HLA-B*0702 and of HLA-C*0702 in the White patients subgroup compared to White controls. To our knowledge, this is the first description of a protective association of HLA-B*0702 and of HLA-C*0702 with SJS with SOC.

**Table 4:** Summary of HLA alleles associated with SJS/TEN with ocular complications.

Allele	Phenotype	Ethnic group	Reference
Negative associations—Protective alleles			
B*0702		British	Present Study
A*1101		Brazilian	^24^
		Japanese	^26^
A*2402	CM SOC	Japanese	^23^
HLA—DQB1*0502		Japanese	^26^
B*0801	SOC	Brazilian	^24^
B*5101	SOC	Brazilian	^24^
B*5701		Indian	^25^
C*0303		Korean	^22^
C*0602		Indian	^25^
Positive associations—Risk alleles			
A*0206	CM SOC	Japanese	^18–21,23,26,27,29,39^
		Korean	^22^
A*3303		Indian	^25^
A*6601	SOC	Brazilian Padro and European ancestry	^17^
	SOC	Brazilian	^24^
B*1301	CM SOC	Japanese	^23^
B*4403	CM SOC	Japanese	^18,23^
	SOC	Indian	^25^
	CM SOC	Thai	^15^
	SOC	Brazilian	^24^
		Indian	^28^
		Brazilian	^28^
		Brazilian of European ancestry	^24,28^
B*5801		Korean	^13^
C*0304	SOC	Korean	^22^
C*0701	SOC	Indian	^25^
C*1403	CM SOC	Japanese	^23^
C*1203	SOC	Brazilian	^24^
		Brazilian of European ancestry	^24^
HLA-DQB1*0601		European	^14^
Haplotypes			
A*3303-C*1403-B*4403	SOC	Japanese	^12^
B*4403-C*0701		Thai	^15^

*CM* Cold medicine, *SOC* severe ocular complications.

Both HLA B*0702, which is prevalent in European populations, and C*0702, which is prevalent in African as well as South African White populations^40^, have been identified as risk alleles in mild, but not severe, cutaneous drug reactions^41–43^. Alfirevic et al*.* 2006 reported frequencies of HLA B*0702 of 18% (15 patients) and 14% (10 patients) in control and mild reaction groups compared to 0% in severe reactions groups^43^. It is however worth noting that within the severe drug reaction subgroup, only two participants were diagnosed with either SJS or TEN. A similar finding in Japanese patients was reported by Ikeda et al*.* associating HLA C*0702 with carbamazepine induced mild cutaneous reactions and absence of HLA C*0702 in their severe group^42^. These data are supported by Moon et al*.* who reported the presence of HLA C*0702 in Korean patients with lamotrigine induced maculopapular eruptions. Alfirevic et al*.* and Ikeda et al. both suggest the presence of these alleles in mild disease and absence in patients with severe disease alludes to a protective role in carbamazepine drug hypersensitivity.

Three non-mutually exclusive mechanisms have been proposed to explain how HLA function is altered in adverse drug reactions including those seen in SJS/TEN^44^:

- The **Hapten/prohapten mechanism** involves the interaction of an offending molecule (drug, metabolite or otherwise) with an endogenous peptide, forming a new immunogenic molecule which by presentation by HLA molecules activates T cell responses and breaks tolerance
- In the **pharmacological interaction model** an offending molecule binds to either the HLA or TCR directly, stimulating a T-cell response.
- In the **altered peptide repertoire model** the offending molecule binds and changes the conformation of the HLA, enabling it to bind a previously unreactive peptide and stimulate a T-cell response.

Furthermore, it is important to consider the dynamics of each of the steps that can lead to variability in such proposed systems. For example, strong HLA-peptide binding dynamics can increase the amount of time a peptide is presented on the cell surface and therefore increase the likelihood of developing a stronger immune reaction to said peptide. This is in turn influenced by peptide pre-processing, which can itself induce variability in HLA-peptide binding. HLA-B*0702 and HLA-C*0702 are associated with strong, dominant responses to Dengue virus and cytomegalovirus respectively, Hosie et al. 2017 and Weiskopf et al. 2013 suggesting a fastidious approach to peptide binding. A peptide- carrier antigen involved in drug reactions with these alleles may not bind effectively which may explain the protective effect of HLA-B*0702 and C*0702 in this study^45,46^.

Thus, our study adds to the increasing body of evidence linking HLA genes to the risk of developing SJS/TEN. This is the first time that HLA B*0702 and C*0702 have been identified as protective alleles for SJS/TEN with SOC in the British population. Critically, these findings demonstrate that protective alleles associated with one phenotype of disease may also be protective in other phenotypes. The reasons for this are uncertain. Patho-mechanistic investigations of the relationships of ethnicity, drug-specificity, phenotype and these alleles will help to understand the regulatory mechanisms of disease expression and the opportunities to develop interventions.

## Supplementary Information

Supplementary Tables.

## Data Availability

The materials and datasets generated during and/or analysed during the current study are available from the corresponding author on reasonable request.
