# Supplementary material for: Human leukocyte antigen B*0702 is protective against ocular Stevens–Johnson syndrome/toxic epidermal necrolysis in the UK population
Source: Sci Rep. 2021 Feb 3;11:2928. doi: 10.1038/s41598-021-82400-3 (PMC7859395; doi:10.1038/s41598-021-82400-3)
Supplement: Supplementary file 1 — Supplementary Tables. [file 41598_2021_82400_MOESM1_ESM.pdf]

Human Leukocyte Antigen B*0702 is protective against Ocular Stevens-Johnson Syndrome/ Toxic Epidermal Necrolysis in the UK population.

Gibran F Butt[1,2], Ali Hassan[3,4], Graham R Wallace[1,2], Shigeru Kinoshita[6], Sajjad Ahmad[3, 4, 5], Mayumi Ueta[6,] and Saaeha Rauz*[1,2]

Supplementary Material

Supplementary Table 1: UK SJS/TEN patient HLA -A, -B and -C typing.

Supplementary Table 2: UK control HLA -A, -B and -C typing.

Supplementary Table 1. UK SJS/TEN patient HLA -A, -B and -C typing.						
	**A (1)**	**A (2)**	**B (1)**	**B (2)**	**C (1)**	**C (2)**
**UK SJS 01**	A*01:01	A*31:01	B*13:02	B*15:24	C*03:03	C*06:02
**UK SJS 02**	A*01:01	A*02:05	B*37:01	B*44:03:01	C*06:02	C*16:01
**UK SJS 03**	A*23:01	A*66:01	B*15:03	B*39:10	C*02:02	C*12:03
**UK SJS 04**	A*01:01	A*30:01	B*13:02	B*44:03:02	C*06:02	C*07:01
**UK SJS 05**	A*31:01	A*68:01	B*35:02	B*40:01	C*03:04	C*04:01
**UK SJS 06**	A*02:11	A*68:01	B*40:06	B*51:01	C*12:02	C*16:02
**UK SJS 07**	A*01:01	A*03:01	B*51:01	B*57:01	C*06:02	C*15:02
**UK SJS 08**	A*01:01	A*02:01	B*40:01	B*44:02	C*05:01	C*07:01
**UK SJS 09**	A*01:01	A*33:03	B*44:03:02	B*44:03:02	C*07:01	C*07:01
**UK SJS 10**	A*02:01	A*11:01	B*44:02	B*51:01	C*03:03	C*05:01
**UK SJS 11**	A*02:01	A*11:01	B*44:03:01	B*51:01	C*14:02	C*16:01
**UK SJS 12**	A*01:02	A*11:01	B*07:02	B*55:01	C*03:03	C*07:02
**UK SJS 13**	A*02:01	A*30:01	B*42:01	B*52:01:02	C*16:01	C*17:01
**UK SJS 14**	A*01:01	A*31:01	B*08:01	B*08:01	C*07:01	C*07:01
**UK SJS 15**	A*02:01	A*02:01	B*07:02	B*08:01	C*07:01	C*07:02
**UK SJS 16**	A*01:01	A*03:01	B*15:17	B*35:01	C*04:01	C*07:01
**UK SJS 17**	A*02:01	A*02:01	B*44:02	B*44:02	C*05:01	C*05:01
**UK SJS 18**	A*02:02	A*02:11	B*15:05	B*51:01	C*03:03	C*16:01
**UK SJS 19**	A*01:01	A*03:01	B*44:02	B*44:02	C*05:01	C*07:04
**UK SJS 20**	A*03:01	A*32:01	B*18:01	B*44:02	C*05:01	C*05:01
**UK SJS 21**	A*11:01	A*24:02	B*13:01	B*44:03	C*04:03	C*07:01
**UK SJS 22**	A*23:01	A*36:01	B*15:03	B*53:01	C*02:02	C*04:01
**UK SJS 23**	A*03:01	A*23:01	B*44:03	B*44:03	C*04:01	C*04:01
**UK SJS 24**	A*02:01	A*02:05	B*40:01	B*44:03	C*03:04	C*16:01
**UK SJS 25**	A*11:01	A*24:02	B*27:04	B*35:01	C*04:01	C*04:01
**UK SJS 26**	A*01:02	A*11:01	B*40:01	B*49:01	C*03:04	C*07:01
**UK SJS 27**	A*01:01	A*02:01	B*07:02	B*57:01	C*06:02	C*07:02
**UK SJS 28**	A*02:01	A*02:01	B*44:02	B*45:01	C*05:01	C*06:02
**UK SJS 29**	A*02:01	A*24:02	B*07:02	B*44:02	C*05:01	C*07:02
**UK SJS 30**	A*11:01	A*23:01	B*15:01	B*44:03	C*04:01	C*12:03
**UK SJS 31**	A*03:01	A*29:02	B*44:02	B*44:03	C*05:01	C*16:01
**UK SJS 32**	A*02:01	A*02:01	B*51:01	B*58:01	C*07:01	C*15:02
**UK SJS 33**	A*02:01	A*02:01	B*08:01	B*44:02	C*05:01	C*07:01

Abbreviations: SJS/TEN, Stevens-Johnson Syndrome/Toxic Epidermal Necrolysis; SOC, Severe ocular complications.

Supplementary Table 2. UK Healthy controls (HC) HLA -A, -B and -C typing.						
	**A (1)**	**A (2)**	**B (1)**	**B (2)**	**C (1)**	**C (2)**
**UK HC 01**	A*01:01	A*24:02	B*08:01	B*15:01	C*03:03	C*07:01
**UK HC 02**	A*30:01	A*32:01	B*13:02	B*14:01	C*06:02	C*08:02
**UK HC 03**	A*03:01	A*03:01	B*07:02	B*35:03	C*04:01	C*07:02
**UK HC 04**	A*02:01	A*02:01	B*18:01	B*44:02	C*05:01	C*07:01
**UK HC 05**	A*30:02	A*33:03	B*07:02	B*15:16	C*14:02	C*15:05
**UK HC 06**	A*01:01	A*24:02	B*08:01	B*51:01	No Pattern	No Pattern
**UK HC 07**	A*01:01	A*01:01	B*57:01	B*57:01	C*06:02	C*06:02
**UK HC 08**	A*11:01	A*68:01	B*35:01	B*35:03	C*04:01	C*12:03
**UK HC 09**	A*02:03	A*11:01	B*18:01	B*52:01	C*07:01	C*07:01
**UK HC 10**	A*01:01	A*23:01	B*07:02	B*14:02	C*07:02	C*08:02
**UK HC 11**	A*03:01	A*26:01	B*07:02	B*45:01	C*06:02	C*07:02
**UK HC 12**	A*01:01	A*03:01	B*07:02	B*08:01	C*07:01	C*07:02
**UK HC 13**	A*01:01	A*24:02	B*07:02	B*35:03	C*04:01	C*07:02
**UK HC 14**	A*11:01	A*34:01	B*38:02	B*40:02	C*07:02	C*15:02
**UK HC 15**	A*02:01	A*02:01	B*08:01	B*18:01	C*07:01	C*07:01
**UK HC 16**	A*03:01	A*11:01	B*07:02	B*27:05	C*02:02	C*07:02
**UK HC 17**	A*03:01	A*30:02	B*58:02	B*78:01	C*06:02	C*16:01
**UK HC 18**	A*01:01	A*02:01	B*07:02	B*08:01	C*07:01	C*08:02
**UK HC 19**	A*02:01	A*29:02	B*44:03	B*47:01	C*06:02	C*16:01
**UK HC 20**	A*02:01	A*30:01	B*13:02	B*44:02	C*05:01	C*06:02
**UK HC 21**	A*25:01	A*29:02	B*44:03	B*44:03	C*16:01	C*16:01
**UK HC 22**	A*02:01	A*26:01	B*07:02	B*27:05	C*01:02	C*07:02
**UK HC 23**	A*02:01	A*11:01	B*18:01	B*44:02	C*05:01	C*07:01

Abbreviations: SJS/TEN, Stevens-Johnson Syndrome/Toxic Epidermal Necrolysis; SOC, Severe ocular complications.
